# Caffeine does not entrain the circadian clock but improves daytime alertness in blind patients with non-24-hour rhythms

**DOI:** 10.1016/j.sleep.2015.01.018

**Published:** 2015-06

**Authors:** Melissa A. St. Hilaire, Steven W. Lockley

**Affiliations:** a Division of Sleep and Circadian Disorders, Departments of Medicine and Neurology, Brigham and Women's Hospital, Boston, MA, USA; b Division of Sleep Medicine, Department of Medicine, Harvard Medical School, Boston, MA, USA; c Faculty of Health and Medical Sciences, University of Surrey, Guildford, Surrey, UK

**Keywords:** Blindness, Non-24-hour sleep–wake disorder, Caffeine, Circadian, Entrainment, Alertness

## Abstract

**Objective/Background:**

Totally blind individuals are highly likely to suffer from Non-24-Hour Sleep-Wake Disorder due to a failure of light to reset the circadian pacemaker in the suprachiasmatic nuclei. In this outpatient case series, we investigated whether daily caffeine administration could entrain the circadian pacemaker in non-entrained blind patients to alleviate symptoms of non-24-hour sleep–wake disorder.

**Patients/Methods:**

Three totally blind males (63.0 ± 7.5 years old) were studied at home over ~4 months. Urinary 6-sulphatoxymelatonin (aMT6s) rhythms were measured for 48 h every 1–2 weeks. Participants completed daily sleep–wake logs, and rated their alertness and mood using nine-point scales every ~2–4 h while awake on urine sampling days. Caffeine capsules (150 mg per os) were self-administered daily at 10 a.m. for approximately one circadian beat cycle based on each participant's endogenous circadian period τ and compared to placebo (*n* = 2) or no treatment (*n* = 1) in a single-masked manner.

**Results:**

Non-24-h aMT6s rhythms were confirmed in all three participants (*τ* range = 24.32–24.57 h). Daily administration of 150 mg caffeine did not entrain the circadian clock. Caffeine treatment significantly improved daytime alertness at adverse circadian phases (*p *<* *0.0001) but did not decrease the occurrence of daytime naps compared with placebo.

**Conclusions:**

Although caffeine was able to improve daytime alertness acutely and may therefore provide temporary symptomatic relief, the inability of caffeine to correct the underlying circadian disorder means that an entraining agent is required to treat Non-24-Hour Sleep–Wake Disorder in the blind appropriately.

## Highlights

• Most totally blind individuals have Non-24-Hour Sleep–Wake Disorder (N24HSWD).
• Optimal treatment should reset the underlying non-entrained circadian pacemaker.
• We tested daily caffeine treatment (150 mg, 10 a.m.) in three blind patients with N24HSWD.
• Caffeine treatment improved daytime alertness at adverse circadian phases.
• Caffeine treatment was unable to entrain the circadian clock.

## 1 Introduction

The circadian pacemaker in the suprachiasmatic nuclei (SCN) of the hypothalamus is synchronized daily to the 24-h light–dark cycle primarily via blue-light-sensitive melanopsin-containing retinal ganglion cells that project to the SCN via the retinohypothalamic tract (RHT) [1]. Most totally blind individuals have a nonfunctional RHT, and thus the circadian clock cannot be entrained by light and reverts to its intrinsic non-24-h period, leading to disruption of circadian rhythms, including melatonin, cortisol, sleep–wake cycles, alertness, and performance [2–4]. The resultant clinical disorder, Non-24-Hour Sleep–Wake Disorder (N24HSWD), is characterized by cyclic episodes of poor nighttime sleep, an increased frequency and duration of daytime sleep, and disruptions in daytime alertness and performance [3,4]. While we and others have shown that daily melatonin administration can entrain the circadian clock in blind individuals [5,6], we aimed to investigate whether caffeine could perform this role. Although a direct phase-shifting effect of caffeine was not demonstrated in one study in mammals [7], several in vitro studies suggest that caffeine can phase-shift the clock [8–11] with a phase response curve similar to that of light [12,13]. We therefore hypothesized that morning caffeine administration (CT1-4) could induce the daily phase advance required to reset the circadian clock in the majority of totally blind people [14], and have direct stimulant benefits on daytime alertness and performance [15,16] while minimizing the negative impact on nighttime sleep.

## 2 Materials and methods

Three blind males (63.0 ± 7.5 years) who were habitual caffeine users (6–15 cups of tea/coffee per day) and had no light perception according to self-report (*n* = 2; S84 and S85) or no eyes (*n* = 1; S33) were studied at home. All participants were healthy and drug-free (Supplementary Table S1). Sequential urine samples were collected every ~4 h (~8 h overnight) for 48 h every 1–2 weeks and assayed for 6-sulphatoxymelatonin (aMT6s), the major urinary metabolite of melatonin and a reliable marker of the circadian clock, by radioimmunoassay [17] (Stockgrand Ltd., University of Surrey, Guildford, UK). Each participant was studied for at least two circadian beat cycles based on circadian period (*τ*) estimates over four weeks of screening (Supplementary Materials). The period of the aMT6s rhythm was assessed using a regression analysis [2] (Origin 8.5 Pro, OriginLab Corporation, Northampton, MA, USA) weighted by the inverse of the squared standard error of the cosinor-derived acrophase (peak) times. Participants completed daily sleep–wake logs (Supplementary Materials) [3,18]. Alertness and mood were assessed using four nine-point scales (alert–sleepy, cheerful–miserable, calm–tense, and depressed–elated) every ~2–4 h while awake on urine sampling days [4].

**Fig. 1:**
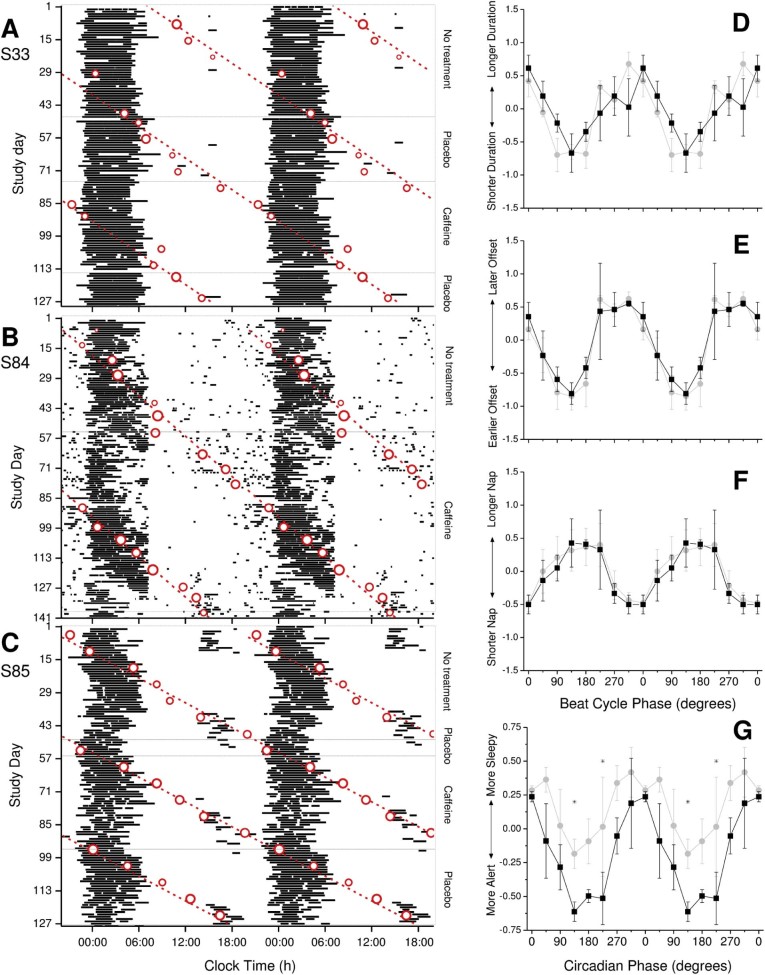
(A–C) Raster double plots of the self-reported sleep times (horizontal black bars), including naps, in totally blind participants S33 (A), S84 (B), and S85 (C). Sequential study days are shown on the ordinate and clock time is double-plotted on the abscissa. Circadian acrophases, which were estimated from cosinor fits to 48-h profiles of aMT6s rhythms, are superimposed (open circles) along with a best-fit regression line (dashed lines) to illustrate the intrinsic non-24-h period. The size of the circle is inversely proportional to the standard error of the circadian phase estimate; the best-fit regression was weighted based on these standard errors. Circadian period was estimated for each condition for each participant. S33: no treatment *τ* = 24.49 ± 0.21 h; placebo *τ* = 24.28 ± 0.27 h; caffeine *τ* = 24.46 ± 0.11 h. S84: no treatment *τ* = 24.25 ± 0.06 h; caffeine *τ* = 24.35 ± 0.02 h. S85: no treatment *τ* = 24.51 ± 0.10 h; placebo *t* = 24.59 ± 0.03 h; caffeine *τ* = 24.59 ± 0.03 h. (D–G) Nighttime sleep duration (D), sleep offset (E), and daytime nap duration (F) plotted as a function of beat cycle phase and alert–sleepy scales (G) plotted as a function of circadian phase during the placebo/no treatment (gray circles) and caffeine (black squares) arms of the study across all three participants. Each parameter was normalized in each participant as the deviation from the mean (*y*-axis; 45° bins), where 0° represents the time at which the midpoint of sleep (for D–F) or the rating assessment (G) coincided with the acrophase of the aMT6s rhythm (*x*-axis) as a function of the treatment condition (placebo/no treatment vs. caffeine). Significant circadian rhythms are indicated by filled symbols and nonsignificant rhythms by open symbols. Phase bins in which caffeine was significantly different from the placebo/no treatment condition are indicated (*).

Caffeine (150 mg fast-release preparation; Martindale Pharmaceuticals, UK) was administered daily at 10 a.m. uninterrupted for approximately one circadian beat cycle in a single-masked design. Caffeine treatment was scheduled to be initiated at CT1-4 at or just after each participant reached a normal circadian phase (ie, aMT6s peak = 04:30 a.m.). Placebo was also administered in S33 and S85 for approximately one circadian beat cycle split before and after caffeine treatment (see Fig. 1); S84 received caffeine only. The study was approved by the University of Surrey Ethics Committee (EC/2003/144/SBMS). Written informed consent was obtained prior to the study and participants were informed that they were free to withdraw at any time.

The mean and standard deviation of each sleep–wake and mood parameter were calculated for each condition for each participant (PROC MEANS, SAS v9.2; SAS Institute, Cary, NC, USA). Statistically significant differences between conditions were calculated using the Wilcoxon Rank-Sum Test (PROC NPAR1WAY, SAS v9.2). The Students' *t*-test was used to assess whether *τ* differed significantly from 24 h, whether participants were entrained to the 24-h day, and to compare *τ* between conditions. A general linear model (PROC GLM in SAS 9.2) was used to compute statistical differences between treatment conditions (placebo/no treatment vs. caffeine) across all individuals with respect to circadian phase [calculated as the difference between the midpoint of the sleep episode (for sleep–wake parameters) or the time of the mood assessment (for alertness/mood) and the aMT6s acrophase divided by the estimated circadian period and multiplied by 360 (degrees), in 45° bins] (*α* < 0.05 after Bonferroni correction). Post-hoc comparisons for significant treatment-by-phase interactions were conducted using PROC MULTTEST (SAS v9.2).

## 3 Results

No participant entrained to 24 h during the caffeine treatment (Fig. 1) and *τ* did not differ from placebo/no treatment based on overlap of the regression 95% confidence intervals (CIs). The non-24-h circadian periods (mean ± 95% CI) over the entire study were 24.44 ± 0.02 h (S33), 24.32 ± 0.01 h (S84), and 24.57 ± 0.01 h (S85), equivalent to beat cycle lengths of 56, 76, and 43 days, respectively. Post-hoc analyses of these circadian periods showed that caffeine treatment was given for 70% (39 days), 111% (84 days), and 91% (39 days), and placebo/no treatment was given for 159% (89 days), 74% (56 days), and 205% (88 days) of the full circadian cycle for S33, S84, and S85, respectively. Furthermore, when all aMT6s acrophases obtained prior to the caffeine condition were included in a post-hoc regression, the *τ* estimates were 24.41 ± 0.07 h, 24.25 ± 0.06 h, and 24.51 ± 0.06 h for S33, S84, and S85, respectively, resulting in the first dose of caffeine being given at ~CT 15.50 for S33, ~CT 21.25 for S84, and ~CT 6.75 for S85 rather than CT1-4 as targeted.

The direct effects of caffeine on sleep were variable across participants, with shorter sleep latencies in two participants compared with placebo/no treatment (*p* = 0.007 and 0.02 for S33 and S85, respectively), but longer latencies during caffeine in S84 (*p* = 0.01) (Table 1). Nighttime and 24-h sleep duration were significantly different across all three conditions for S33 (*p* = 0.02 for both). Post-hoc comparisons showed significant differences between the no treatment and placebo conditions (*p* = 0.03 and 0.01, respectively): total sleep amounts were lower during no treatment, which coincided with an adverse circadian phase (Fig. 1A). Although sleep offset was significantly different across conditions for S33 (*p* = 0.03), post-hoc comparisons revealed no pairwise differences (Table 1).

As expected, significant circadian rhythms were observed in nighttime awakening duration, nighttime sleep duration (Fig. 1D), sleep offset (Fig. 1E), number of naps, and duration of naps (Fig. 1F) (both placebo/no treatment and caffeine, all *p *<* *0.03) and sleep onset and sleep quality (caffeine only, *p* = 0.0006 and 0.0005, respectively), but no significant main effect of treatment or treatment-by-phase interaction was observed for any sleep–wake parameters.

Alertness and mood changes were not consistent across participants (Table 1); caffeine improved alertness and cheerfulness in S33, and S84 and S85 rated themselves calmer during caffeine treatment (Table 1). A significant circadian rhythm was observed in alert–sleepy scores for both the placebo/no treatment (*p* = 0.04) and caffeine (*p* = 0.0004) conditions when averaged across participants, with peak sleepiness occurring during the biological night at the aMT6s acrophase (Fig. 1G). There was a significant group-alerting effect of caffeine (*p* = 0.0005), with interaction effects at 135° (*p* = 0.03) and 225° (*p* = 0.002), equivalent to ~1.30 p.m. and ~7.30 p.m., respectively, under normal entrainment (ie, sleep at night, awake during the day); caffeine significantly increased alertness when participants were awake at these adverse circadian phases. A similar trend was observed for the circadian rhythm in cheerful–miserable assessments (*p* = 0.07); participants rated themselves more cheerful during caffeine treatment. No significant circadian rhythms were observed in the calm–tense or depressed–elated scores.

## 4 Discussion

Daily administration of 150 mg of caffeine was unable to entrain the non-24-h rhythms in any of the three totally blind individuals studied. These results indicate that a daily 150 mg dose of caffeine at 10.00 a.m. is not effective as a circadian entraining agent. Our results do show, however, that the morning administration of caffeine directly mitigates some of the negative impact of non-entrained rhythms on daytime alertness and mood, without addressing the underlying circadian disorder.

The strength of this study is that the circadian effects of caffeine were studied in non-entrained individuals and in the absence of light, considered a gold-standard approach for assessing circadian rhythm entrainment. Although we cannot exclude the possibility that caffeine had a small resetting effect at some phases of administration that exceeded the limit of detectability in this study, we can state definitively that caffeine at this dose and duration of administration did not entrain the circadian clock, which was the primary aim of the experiment, as evidenced by a failure to observe a change in the intrinsic circadian period during the caffeine phase of the protocol. While the study was limited to a small number of cases, the distinct non-entrained phenotype would have permitted clear evidence of entrainment, if present, at this single dose and preparation even with this limited number of participants. The response to other doses may vary, however, and we did not screen for interindividual differences in caffeine sensitivity (eg, ADORA2A gene polymorphisms [19,20]). Despite exposure to multiple additional non-photic time cues (eg, activity, meal timing, exercise, and alcohol) [21,22], there was also no evidence of entrainment to the 24-h day during no treatment and placebo conditions, consistent with our previous work [2,5,23].

Although we attempted to initiate caffeine treatment at the same circadian phase across participants, post-hoc analysis of the initial treatment phase showed that it was not consistent (Fig. 1). Administering caffeine for most or all of a circadian beat cycle ensured that caffeine was administered during both the advance and delay phases of any theoretical phase response curve, however, thereby mitigating this confound. Even among participants who did not receive treatment for a full beat cycle (S33 and S85), all participants received treatment for almost all the phase advance portion of the phase response curve. If the caffeine phase response curve is similar to that of light as we assumed [12,13], then we would expect to see evidence of phase advances, such as a shortened circadian period, even in the absence of full entrainment, which we did not observe [23]. In addition, although we used sleep–wake logs rather than actigraphy recordings to measure nocturnal sleep and daytime naps, prior studies in blind individuals [3,18] have reported a good correlation between the two methods to measure the timing and duration of the sleep episode (*r* = 0.48–0.88) [18].

In conclusion, daily administration of 150 mg caffeine failed to entrain the circadian pacemaker in totally blind patients. Caffeine was able to increase alertness and mood directly, however, and may therefore be a useful adjunct therapy to provide temporary relief for the sleepiness symptoms of N24HSWD in the absence of appropriate treatment with a circadian regulator.

## Conflict of interest

Neither author has any conflicts of interest directly associated with the current study. Dr. Lockley was the principal investigator of two recently completed and one ongoing sponsored clinical trials of a melatonin agonist for the treatment of non-24-hour sleep–wake disorder in the blind, sponsored by Vanda Pharmaceuticals. Inc., and has received an investigator-initiated research grant and two service agreements from Vanda Pharmaceuticals, Inc., related to non-24-hour rhythms in the blind. He has also received minor consulting fees from 14 financial companies related to non-24-hour sleep–wake disorder in the blind and the publicly available clinical trial results. He has also received honoraria from MediCom Worldwide, Inc., for teaching on a CME course sponsored by Vanda Pharmaceuticals, Inc.; for contributing text about non-24-hour sleep–wake disorder for the National Sleep Foundation and textbook chapters published by Elsevier; and in 2007 received an authorship fees from Servier Inc., for writing a review of circadian rhythm disorders in the blind.

Dr. Lockley also reports receiving consulting fees in the past from American Family, Apollo Lighting, Brigham and Women's Hospital, Naturebright, Thomas Jefferson University, Warwick Medical School, and Wyle Integrated Science and Engineering and Wyvern Funds; and holds current consulting contracts with Headwaters, PlanLED and Wyle Integrated Science and Engineering; unrestricted equipment gifts from Bioilluminations LLC, Bionetics Corporation, and ResMed Inc.; an unrestricted monetary gift to support research from Swinburne University of Technology, Australia; a fellowship gift from Optalert, Pty, Melbourne, Australia; equity in iSLEEP, Pty, Melbourne, Australia; advance author payment and royalties from Oxford University Press; honoraria for written articles by AMO Inc., and the Wall Street Journal; honoraria plus travel, accommodation or meals for invited seminars, conference presentations or teaching from 2nd International Symposium on the Design of Artificial Environments; American Society for Photobiology; Bassett Research Institute; Brookline Adult Education; Brown University; Emergency Social Services Association Conference; Harvard University (CME); I Slept Great/Euforma, LLC; International Graduate School of Neuroscience; Japan National Institute of Occupational Safety and Health; Lightfair; North East Sleep Society; Notre Dame University; Takeda Pharmaceuticals North America; Thomas Jefferson University; University of Vermont College of Medicine; Velux; travel and accommodation support (no honoraria) for invited seminars, conference presentations or teaching from 8th International Conference on Managing Fatigue; 14th Annual Tennessee Perfusion Conference; American Academy of Sleep Medicine; Apollo Lighting; Bar Harbor Chamber of Commerce; Canadian Sleep Society; Committee of Interns and Residents; Coney Island Hospital; Connecticut Business & Industry Association Health and Safety Conference; Emergency Services Steering Committee; FASEB; Ferrari; Harvard University; Illinois Coalition for Responsible Outdoor Lighting; Lighting Science Group Corp; Massachusetts General Hospital; National Research Council Canada; New England College of Occupational and Environmental Medicine; New York Academy of Sciences; Ontario Association of Fire Chiefs; Oxford University; Philips Lighting; Rio Tinto; Sleep HealthCenters; UMass Memorial; University of Manchester; University of Montreal; University of Texas Medical Branch; University of Tsukuba; Vanda Pharmaceuticals Inc.; Warwick Medical School; Woolcock Institute of Medical Research; Wyle Integrated Science and Engineering (NASA). Dr. Lockley has completed investigator-initiated research grants from Alcon Inc, and Apollo Lighting and has ongoing investigator-initiated research grants from Biological Illuminations LLC, and Respironics Inc., has a service agreement with Rio Tinto Iron Ore; and has received two investigator-initiated research grants from the ResMed Foundation. Dr. Lockley holds a process patent for the use of short-wavelength light for resetting the human circadian pacemaker and improving alertness and performance which is assigned to the Brigham and Women's Hospital per Hospital policy. He has also received minor revenue from a patent on the use of short-wavelength light which is assigned to the University of Surrey. Dr. Lockley has also served as a paid expert on behalf of six public bodies and one union on arbitrations related to sleep, circadian rhythms, and work hours.

The ICMJE Uniform Disclosure Form for Potential Conflicts of Interest associated with this article can be viewed by clicking on the following link: http://dx.doi.org/10.1016/j.sleep.2015.01.018.

Conflict of interest ICMJE Form for Disclosure of Potential Conflicts of Interest form.

## Figures and Tables

**Table 1:** Average sleep and mood parameters by participant across placebo and caffeine conditions.

	S33				S84			S85			
	No treatment	Placebo	Caffeine	*p*	No treatment	Caffeine	*p*	No treatment	Placebo	Caffeine	*p*
Sleep onset (hh:mm)	23:08 ± 00:50	23:10 ± 00:30	23:01 ± 00:35	0.14	23:42 ± 00:39	23:53 ± 01:04	0.89	23:26 ± 00:57	23:10 ± 00:46	23:01 ± 00:47	0.07
Sleep latency (min)	1.63 ± 2.91	1.61 ± 3.64	0.13 ± 0.80	***0.007***	16.86 ± 15.77	35.36 ± 61.57	***0.01***	15.53 ± 8.36	15.39 ± 6.08	11.81 ± 2.44	***0.02***
Sleep offset (hh:mm)	05:36 ± 01:11	06:03 ± 01:09	05:49 ± 00:59	***0.03***	05:52 ± 01:12	05:57 ± 01:36	0.07	04:54 ± 01:21	05:06 ± 01:27	04:52 ± 01:29	0.63
WASO (number/night)	0.92 ± 1.13	1.02 ± 1.04	0.69 ± 0.77	0.37	3.46 ± 1.08	3.12 ± 1.27	0.11	0.82 ± 0.73	0.97 ± 0.74	1.36 ± 0.84	***0.005***
WASO duration (min)	14.98 ± 39.78	9.27 ± 38.69	9.74 ± 21.97	0.33	179.24 ± 69.13	208.06 ± 89.60	0.07	19.16 ± 32.66	28.03 ± 34.45	33.49 ± 29.98	***0.006***
Sleep quality	1.65 ± 1.63	1.49 ± 1.40	1.28 ± 1.00	0.48	6.93 ± 1.14	7.06 ± 1.17	0.74	3.44 ± 1.12	3.03 ± 0.87	3.15 ± 0.81	0.17
Nighttime sleep duration (min/night)	372.73 ± 64.73	403.83 ± 68.76	398.97 ± 64.11	***0.02***	352.38 ± 74.19	336.25 ± 117.97	0.80	308.55 ± 83.90	328.41 ± 88.71	317.62 ± 78.16	0.56
Naps (number/day)	0.08 ± 0.35	0.10 ± 0.30	0.00 ± 0.00	0.16	1.64 ± 1.43	2.13 ± 1.95	0.21	0.47 ± 0.58	0.32 ± 0.47	0.28 ± 0.46	0.26
Nap duration (min)	2.98 ± 12.50	7.32 ± 23.98	0.00 ± 0.00	0.15	25.35 ± 23.02	39.16 ± 35.33	0.05	41.94 ± 60.46	27.59 ± 45.11	27.18 ± 48.03	0.35
24-h sleep duration (min/24 h)	368.96 ± 85.14	411.15 ± 72.93	398.97 ± 64.11	***0.02***	382.07 ± 71.85	376.31 ± 115.33	0.61	352.53 ± 82.23	356.00 ± 66.64	344.79 ± 78.69	0.91
Alert (1)–Sleepy (9)	1.51 ± 0.51	1.39 ± 0.63	1.09 ± 0.29	***<0.0001***	5.84 ± 1.78	5.52 ± 1.94	0.14	3.72 ± 1.62	3.64 ± 1.38	3.36 ± 1.07	0.53
Cheerful (1)–Miserable (9)	1.12 ± 0.33	1.17 ± 0.51	1.00 ± 0.00	***0.04***	5.01 ± 1.33	4.91 ± 1.34	0.42	2.75 ± 0.66	2.80 ± 0.52	2.72 ± 0.57	0.31
Calm (1)–Tense (9)	1.00 ± 0.00	1.05 ± 0.21	1.00 ± 0.00	0.13	4.40 ± 1.61	3.92 ± 1.36	***0.003***	2.65 ± 0.48	2.79 ± 0.41	2.85 ± 0.36	***0.008***
Depressed (1)–Elated (9)	5.00 ± 0.00	5.00 ± 0.00	5.00 ± 0.00	1.00	4.71 ± 0.89	4.83 ± 0.81	0.14	5.07 ± 0.33	5.00 ± 0.00	5.00 ± 0.00	***0.03***
