# Supplementary material for: Caffeine does not entrain the circadian clock but improves daytime alertness in blind patients with non-24-hour rhythms
Source: Sleep Med. 2015 Jun;16(6):800–4. doi: 10.1016/j.sleep.2015.01.018 (PMC4465963; doi:10.1016/j.sleep.2015.01.018)
Supplement: Appendix S1 — Table S1 and Fig. S1. [file mmc2.pdf]

Supplementary Materials

Beat cycle calculation

A beat cycle was defined as the number of calendar days required for the circadian phase to complete one circadian cycle (τ) (e.g., if τ = 24.5 h, then phase changes by 0.5 h/day. It would take 49 days to cycle through 24.5 hours. The beat cycle is calculated from [24 / (τ - 24)] +1). τ was assessed using a regression analysis (Origin 8.5 Pro; OriginLab Corporation, Northampton, MA, USA) weighted by the inverse of the squared standard error (i.e., instrumental weighting) of the cosinor-derived acrophase times from the aMT6s rhythm. To calculate τ, 24 was added to the slope of the best-fit regression line. The estimate of τ during the pre-treatment/screening phase (e.g., the first ~4 weeks) of the study was used to determine the duration of the experiment for each subject. The estimate of τ over the first ~4 weeks of the study was 24.60 h (equivalent to a beat cycle of 41 days) for S33, 24.23 h (equivalent to a beat cycle of 105 days) for S84, and 24.61 h (equivalent to a beat cycle of 40 days) for S85. These τ and beat cycle estimates were used to time the duration and timing of caffeine and placebo administration in each participant. Note that these τ and beat cycle estimates obtained *a priori* from the first 4 weeks of screening are different from the τ and beat cycle estimates reported in the **Results**, which were determined from a post hoc analysis that included acrophases across the entire study.

Sleep-Wake Logs

Participants were asked to complete the sleep-wake log each morning over the duration of the study. Participants were asked to record the clock time at which they got into bed the previous night, the clock time at which they tried to fall asleep, the clock time at which they fell asleep (i.e., the sleep onset), the clock times of any awakenings throughout the nighttime sleep episode (both start and end times), the clock time of their final awakening (i.e., the sleep offset), the clock time at which they got out of bed, and sleep quality (scored on a 1-9 scale, 1 = best sleep ever, 9 = worst sleep ever). The participants also reported the start and end times of any nap episodes that occurred in the previous 24 hours. The following self-reported sleep and wake parameters were analyzed: sleep onset (in clock time), sleep latency (calculated as the difference between sleep onset and the time at which the participant attempted to fall asleep, in mins), number of awakenings after sleep onset, duration of awakenings after sleep onset (in mins), sleep quality (1-9), nighttime sleep duration (calculated from the difference between the sleep onset and the sleep offset minus the duration of nighttime awakenings, in mins), sleep offset (in clock time), number of naps, duration of naps (in mins), and total 24-hr sleep duration (which was calculated as the sum of the nighttime sleep duration and the number of naps ± 12 hours of the estimated acrophase.

Supplementary Table

Table S1: Participant medical and demographic information.

	**S33**	**S84**	**S85**
**Age at start of study**	63 years old	69 years old	55 years old
**Age of onset of blindness**	7 years old	10 years old	Birth
**Medical diagnosis**	Accident	Accident **-** burst left eye ball	Retinopathy of prematurity
**Number of eyes present**	0	1 (wears prosthesis over right eye)	2 (no light perception in either eye)
**Occupation**	Retired	Retired	Unemployed
**Caffeine use (pre-study)**	15 cups of tea/day	6 cups of tea or coffee/day	8 cups of tea/day
**Alcohol use (during study)**	0.21 ± 0.57 units/day	2.01 ± 0.99 units/day	0 units/day
**Tobacco use (during study**	Non-smoker	Non-smoker	Non-smoker
**Medication use (pre-study)**	None	Occasional sleeping pill	None
**Medication use (during study)**	N/A	N/A	N/A
**Average exercise/activity (during study)**	0 hours/day	1.58 ± 2.17 hours/day (walking)	0.29 ± 0.44 hours/day (walking)

Supplementary Figure

Figure S1: (A-C) Raster double-plots of the self-reported sleep times (horizontal black bars) and in-bed times (horizontal open bars), including naps, in totally blind participants S33 (**A**), S84 (**B**), and S85 (**C**). As in **Figure 1**, sequential study days are shown on the ordinate and clock time is double-plotted on the abscissa. Circadian acrophases, which were estimated from cosinor fits to 48-hr profiles of aMT6s rhythms, are superimposed (open circles) along with a best-fit regression line (dashed lines) to illustrate the intrinsic non-24-hour period. The size of the circle is inversely proportional to the standard error of the circadian phase estimate; the best-fit regression was weighted based on these standard errors. The circadian period of each condition for each participant is reported in the figure legend for **Figure 1**.

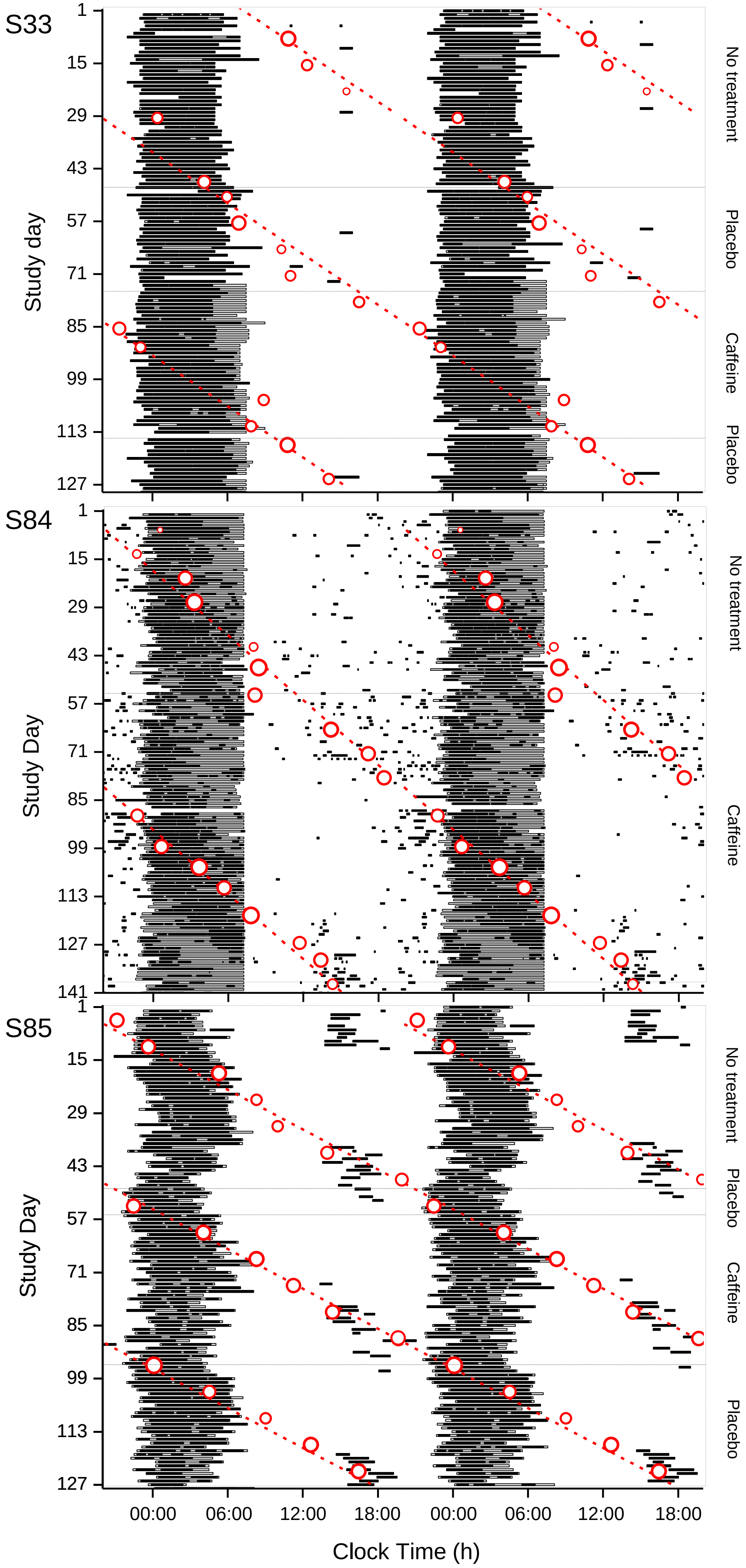

S33
S84
S85
Study day
Study Day
Study Day
1
15
29
43
57
71
85
99
113
127
141
No treatment
Placebo
Caffeine
Placebo
No treatment
Caffeine
No treatment
Placebo
Caffeine
Placebo
00:00
06:00
12:00
18:00
00:00
06:00
12:00
18:00
Clock Time (h)
